# Correction: Impact of post-licensure radiation safety training on catheterization laboratory radiation practices among interventional cardiologists in India: a nationwide survey

**DOI:** 10.3389/fpubh.2026.1933061

**Published:** 2026-07-20

**Authors:** Frontiers Production Office

**Affiliations:** Frontiers Media SA, Lausanne, Switzerland

**Keywords:** catheterization laboratory, interventional cardiology, occupational exposure, radiation protection, radiation safety training

There was a missing figure in the published article. The **Graphical Abstract** was missed to be included. The corrected figure appears below:

**Graphical Abstract:**
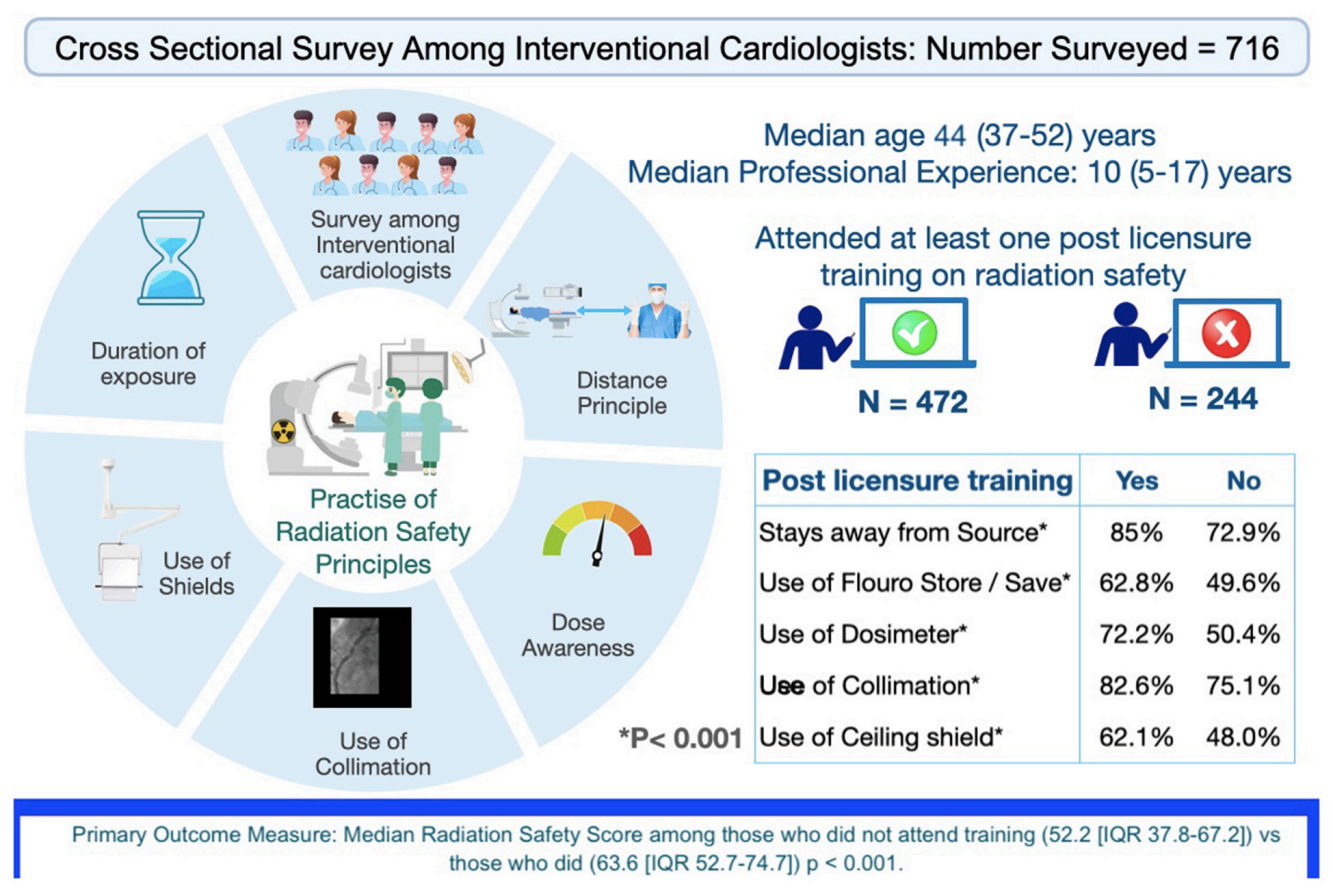


The original version of this article has been updated.

## Generative AI statement

Any alternative text (alt text) provided alongside figures in this article has been generated by Frontiers with the support of artificial intelligence and reasonable efforts have been made to ensure accuracy, including review by the authors wherever possible. If you identify any issues, please contact us.

