# Supplementary material for: Impact of post-licensure radiation safety training on catheterization laboratory radiation practices among interventional cardiologists in India: a nationwide survey
Source: Front Public Health. 2026 Jun 22;14:1808498. doi: 10.3389/fpubh.2026.1808498 (PMC13333479; doi:10.3389/fpubh.2026.1808498)
Supplement: Supplementary file 1 [file Supplementary_File_1.pdf]

Radiation Safety Awareness in the Cathlab - Questionnaire

1. How old are you?
2. Which Gender you belong to? (Male / Female / Prefer not to say)
3. What is your field of specialization? (Options: Interventional Cardiologist, Electrophysiologist, Pediatric Cardiologist, Interventional Radiologist, Cath lab technician, Cath lab staff nurse, Other)
4. Have you attended a lecture on radiation safety or a course on radiation safety anytime before? (Yes / No)
5. For how many years are you exposed to radiation in cardiology?
6. Do you have a personal dosimeter badge? (Yes / No)
7. Do you monitor your radiation dose? (Always / Sometimes / Never)
8. Do you use ceiling suspended lead glass shields? (Always / Most of the time / Rarely / Never / Facility not available)
9. Do you use under-table shields? (Always / Most of the time / Rarely / Never / Facility not available)
10. Do you use radpads or similar disposable radiation shields? (Always / Most of the time / Rarely / Never / Facility not available)
11. Do you use lead goggles for the eye? (Always / Most of the time / Rarely / Never / Facility not available)
12. Do you wear lead cap for head? (Always / Most of the time / Rarely / Never / Not available)
13. Do you wear thyroid collar? (Always / Most of the time / Rarely / Never / Facility not available)
14. Do you wear shin / leg guards? (Always / Most of the time / Rarely / Never / Facility not available)
15. Do you use fluorostore / fluorosave instead of cine? (Always / Most of the time / Rarely / Never / Not available)
16. Do you use 7.5 frames/sec or lower? (Yes / No / Maybe)

17. Do you have any medical problem which you feel is related to radiation exposure? (Yes / No)
18. Do you have neck pain / back pain? (Yes / No)
19. Do you have cataract of the eyes? (Yes / No / I don't know)
20. What frame rate do you use for coronary angiogram? (15 / 7.5 / 3.8 / I don't know)
21. What frame rates do you use for pacemaker implant? (15 / 7.5 / I don't know / Other)
22. Do you give radiation dose used in your procedure report? (Yes / No)
23. What is the make of your cath lab machine?
24. What is your name?
25. What is the name of your institute?
26. Please share your email ID and Phone number.
27. Which state/UT in India are you practicing? (Multiple options including Outside India)
28. Do you insist on proper collimation during fluoroscopy and cine? (Always / Most of the time / Rarely / Never / I don't know about collimation)
29. How often is your cath lab calibrated for radiation safety? (Once a year / Once in six months / Never / I don't know / Other)
30. Do you ensure the detector is kept close to the patient's body? (Always / Most of the time / Rarely / Never)
31. Do you move away from the X-ray tube, when you are not the operator, during cine / fluoro? (Always / Most of the time / Sometimes / Rarely)
32. What is your opinion about radiation safety talks/sessions in cardiology conferences? (Not needed / Needed for PG sessions / Needed for all delegates / Online certificate courses may be organised / Other)
